# Correlation of laminin subunit alpha 3 expression in pancreatic ductal adenocarcinoma with tumor liver metastasis and survival

**DOI:** 10.2478/raon-2024-0020

**Published:** 2024-03-07

**Authors:** Yueyi Xing, Xue Jing, Gong Qing, Yueping Jiang

**Affiliations:** Qingdao University, Qingdao, Shandong Province, China; Gastroenterology Department, the Affiliated Hospital of Qingdao University, Qingdao, Shandong Province, China

**Keywords:** pancreatic ductal adenocarcinoma, laminin subunit alpha 3, liver metastasis, prognosis

## Abstract

**Background:**

The high mortality rate of pancreatic ductal adenocarcinoma (PDAC) is primarily attributed to metastasis. Laminin subunit alpha 3 (LAMA3) is known to modulate tumor progression. However, the influence of LAMA3 on liver metastasis in PDAC remains unclear. This study aimed to elucidate whether LAMA3 expression is increased in PDAC with liver metastasis.

**Patients and methods:**

We extracted information related to LAMA3 expression levels and associated clinicopathological parameters from The Cancer Genome Atlas (TCGA) and four Gene Expression Omnibus (GEO) datasets. Clinicopathological analysis was performed; the Kaplan-Meier Plotter was used to evaluate LAMA3’s prognostic effect in PDAC. We retrospectively collected clinicopathological data and tissue specimens from 117 surgically treated patients with PDAC at the Affiliated Hospital of Qingdao University. We assessed LAMA3 expression and investigated its correlation with the clinicopathological traits, clinical outcomes, and hepatic metastasis.

**Results:**

Amplified expression of LAMA3 was observed in PDAC tissue compared with normal tissue in the TCGA and GEO databases. High LAMA3 expression was associated with poor overall survival (OS) and relapse-free survival (RFS) in patients with PDAC. LAMA3 expression was significantly enhanced in PDAC tissues than in adjacent tissues. Tumor tissues from patients with PDAC exhibiting liver metastasis showed higher LAMA3 expression than those without liver metastasis. High LAMA3 expression correlated with large tumor size and TNM stage. LAMA3 expression and liver metastasis were independent predictive factors for OS; the former was independently associated with liver metastasis.

**Conclusions:**

LAMA3 expression is elevated in patients with PDAC with liver metastasis and is a predictor of prognosis.

## Introduction

Pancreatic ductal adenocarcinoma (PDAC), a disease that is prevalently observed within the digestive system, is distinguished by its severe malignancy and exhibits a disconcerting confluence of incidence and mortality.^1^ The 5-year survival rate of patients with PDAC is < 10%, with an extremely poor prognosis. If this trend is sustained, the impending decade may witness pancreatic cancer ascending to the rank as the second most lethal cancer.^2^ Most patients with PDAC remain asymptomatic until the disease reaches advanced stages. Ninety percent of patients with PDAC diagnosed only after metastasis have a poor prognosis, with 50% developing systemic metastasis.^3,4^ The potential for enduring survival among patients with PDAC considerably depends on tumor size and disease stage. Therefore, early detection of potentially curable cancers is crucial for reducing mortality rates among patients with PDAC. The elucidation of key molecular mechanisms and prospective intervention targets associated with pancreatic cancer metastasis will aid in deciphering the genetic and molecular underpinnings of this disease, provide biomarkers for preliminary warning and metastasis surveillance, and pave the way for enhancing the survival prospects of patients with pancreatic cancer.

Laminin, a heterotrimeric molecule consisting of α, β, and γ subunits, is the primary constituent of the extracellular matrix while collagen and fibronectin form the basement membrane. Among the three subunits of laminin, the α subunit is involved in tissue-specific distribution and biological activity.^5^ Laminin subunit alpha 3 (LAMA3), which encodes for the laminin α subunit, enables its globular carboxyl-terminal domain to engage with integrins at the plasma membrane, thereby participating in intracellular signal transduction.^6^ Currently, LAMA3 contributes to cell proliferation and apoptosis in diverse malignant tumors and modulates tumor progression through signal transduction pathways, such as focal adhesion plaques.^7, 8, 9^ The aberrant expression of LAMA3 in various tumors is inextricably associated with the clinical stage, tumor size, and pathological manifestations of patients.^10^ However, the influence of LAMA3 on liver metastasis in PDAC remains unclear. This study aimed to clarify LAMA3 expression in PDAC and investigate the relationship between LAMA3 expression and liver metastasis in patients with unresectable PDAC.

## Patients and methods

### Procurement of bioinformatics analysis data

RNA sequencing expression traits, along with their associated clinical data pertaining to LAMA3, were procured from the The Cancer Genome Atlas (TCGA) dataset (https://portal.gdc.com). The current-release GTEx datasets were accessed from the GTEx data portal website (https://www.gtexportal.org/home/datasets). The data comprised 179 tumor samples and 4 normal samples sourced from the TCGA, in addition to 328 normal mRNA expression data points from GTEx. Corresponding platform annotation files were obtained from the Gene Expression Omnibus (GEO) database (http://www.ncbi.nlm.nih.gov/geo/) to validate the expression levels of LAMA3 in PDAC. Finally, we identified four datasets: GSE28735 (n = 90), GSE62452 (n = 130), GSE101448 (n = 43), and GSE62165 (n = 131). To perform a clinicopathological analysis of LAMA3, we used University of ALabama at Birmingham CANcer (UALCAN) (http://ualcan.path.uab.edu). Survival curves were generated using the Kaplan-Meier Plotter database (http://kmplot.com/analysis/).^11^

### Acquisition of human pancreatic ductal adenocarcinoma (PDAC) specimens and clinicopathological data

Our study included 117 patients with PDAC who underwent pancreatic surgery at the Affiliated Hospital of Qingdao University. These patients had not received any anticancer treatment before surgery, and the diagnosis of pancreatic carcinoma was confirmed by postoperative pathology. Paraffin-embedded tumor tissues were obtained from each patient, and the corresponding para-carcinomatous tissues were obtained from 60 patients. All patients provided informed consent, and the investigation was conducted in accordance with the Declaration of Helsinki with the endorsement of the Medical Ethics Committee of the Affiliated Hospital of Qingdao University (QYFYWZLL27485 and QYFYWZLL27608).

Clinicopathological data were obtained from retrospective medical records, which consisted of age, sex, tumor size, tumor location, histological grade, perineural invasion, lymph node metastasis, vascular invasion, liver metastasis, tumor-node-metastasis (TNM) stage, preoperative serum carcinoembryonic antigen (CEA), and carbohydrate antigen 19-9 (CA19-9) concentrations. Overall survival (OS) was calculated as the interval between surgery and either death or last follow-up appointment. The dates of death were ascertained from hospital records or follow-up telephone interviews.

### Immunohistochemistry

Paraffin-embedded PDAC and para-cancerous tissues underwent sequential sectioning at a thickness of 4 μm. After baking, deparaffinizing, and hydrating, the paraffin sections were ensconced in a pressure cooker for 10 min for antigen repair. Subsequently, the antigen repair box was relocated to an ice box for a 25-min interval, permitting cooling to room temperature. To curb endogenous peroxidase activity, the tissue sections were immersed in a concoction of 3% hydrogen peroxide and methanol for 15 min. Each section received a blockade of 10% sheep serum and incubated at 37°C for half an hour. This was followed by an overnight incubation at 4°C with primary antibodies (1:100 L, no. ab242197; Abcam Inc.), followed by incubation with secondary antibodies (no. ab242197; Abcam Inc.) at 37°C for 30 min. The tissue sections were then stained with 3, 3-diaminobenzidine (Roche) for 5–10 min at room temperature. Hematoxylin (Roche) was used for counterstaining for 25 s before proceeding with dehydration, clarification, and sealing. Microscopic visualization was performed to record the images. An independent duo of pathologists evaluated all samples.

The cytoplasmic staining score (CF) was defined as follows: 0 (0–20%), 1 (21–50%), 2 (51–75%), and 3 (>75%). Moreover, the cytoplasmic staining intensity (CI) was categorized as 0 (negative), 1–2 (weak), and 3 (strong). The cytoplasmic composite score was calculated as CF×CI.

### Statistical analyses

For all the TCGA and GEO databases, we used the Wilcox test to perform differential expression analysis between tumor and normal tissues. Categorical variables are expressed as frequencies and percentages, and significance was determined using the χ^2^ or Fisher’s exact test. Quantitative variables are expressed as means±standard deviations, and significance was determined using Student’s t-test. Non-normally distributed variables are expressed as medians and interquartile ranges, and significance was determined using the Mann–Whitney U test. Multivariate logistic regression analyses were performed to identify the independent risk factors for PDAC. We used the cutoff points of the test variables produced on receiver operating characteristic curves. Survival analysis was performed using Kaplan–Meier analysis and assessed using the log-rank test. Cox regression analysis was performed to analyze the effect of OS on the survival of patients with PDAC. All analyses were performed using SPSS version 24.0, GraphPad Prism version 8.0.1, and R software version 4.0.3. *P* < 0.05 was considered statistically significant.

## Results

### Elevation of laminin subunit alpha 3 in PDAC and its correlation with prognosis using bioinformatics analysis

**FIGURE 1.**
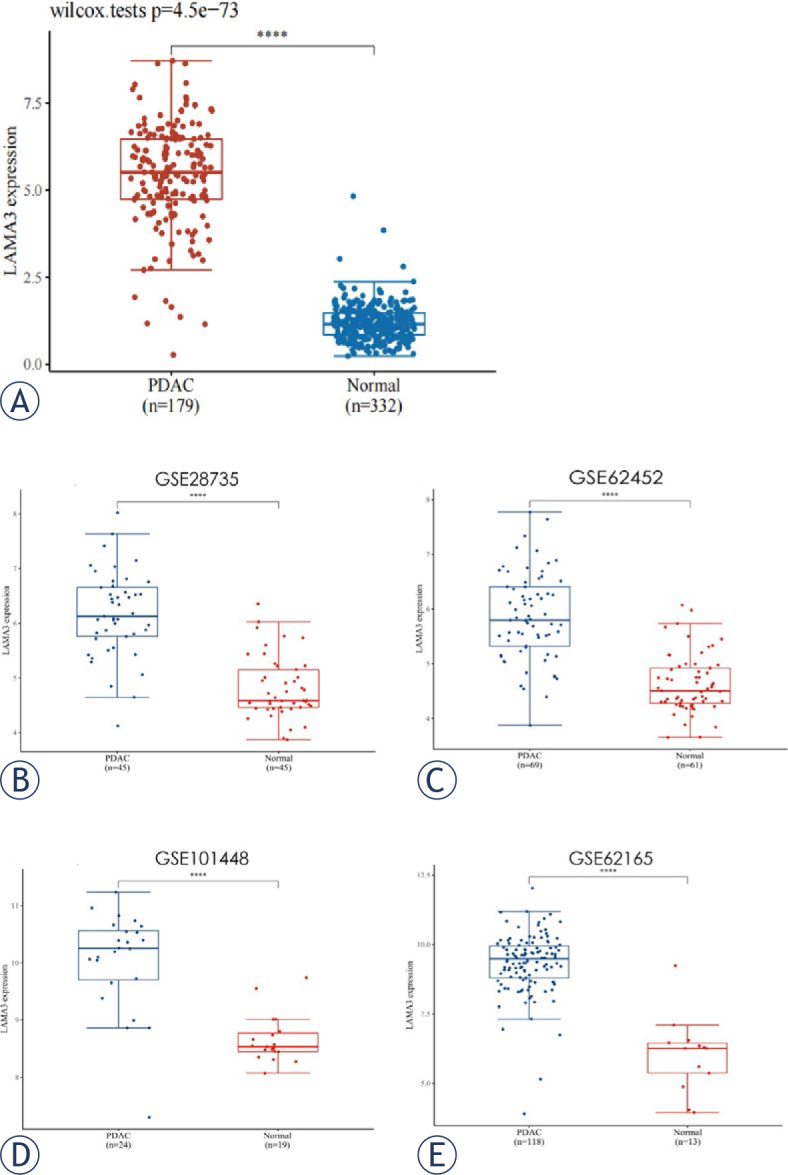
Expression of laminin subunit alpha 3 (LAMA3) in pancreatic ductal adenocarcinoma (PDAC) and normal tissues from the Cancer Genome Atlas (TCGA) database **(A)** and the Gene Expression Omnibus (GEO) database **(B–E)**. Expression of LAMA3 in 179 PDAC and 332 normal tissues from TCGA **(A)**; Expression of LAMA3 in 45 PDAC and 45 normal tissues from GSE28735 cohort **(B)**; Expression of LAMA3 in 69 PDAC and 61 normal tissues from GSE62452 cohort **(C)**; Expression of LAMA3 in 24 PDAC and 19 normal tissues from GSE101448 cohort **(D)**; Expression of LAMA3 in 118 PDAC and 13 normal tissues from GSE62165 cohort **(E)**. ^****^P < 0.001.

Using the TCGA database, we identified a prominent divergence in LAMA3 expression between PDAC tissues (n = 179) and normal tissues (n = 332) (*P* < 0.001) (Figure 1A). Four datasets (GSE28735, GSE62452, GSE101448, and GSE62165) were obtained from the GEO database and used as validation sets. The results showed that LAMA3 was significantly upregulated in PDAC tissues (all *P* < 0.001) (Figure 1B–E).

To further elucidate the role of LAMA3 in PDAC, we investigated its expression using various clinicopathological parameters. LAMA3 expression displayed no remarkable correlation with age (Supplementary Figure 1A), sex (Supplementary Figure 1B), and drinking habits (Supplementary Figure 1C) in patients with PDAC. Grade 1 indicated a well-differentiated (low-grade) tumor, grade 2 denoted a moderately differentiated (intermediate-grade) tumor, grade 3 indicated a poorly differentiated (high-grade) tumor, and grade 4 indicated an undifferentiated (high-grade) tumor. The grade of patients with PDAC influenced LAMA3 expression, and heightened expression was observed in grades 2 and 3 (Supplementary Figure 1D). However, there was no significant difference in LAMA3 expression with respect to nodal metastasis (Supplementary Figure 1E) or diabetes (Supplementary Figure 1F).

**FIGURE 2.**
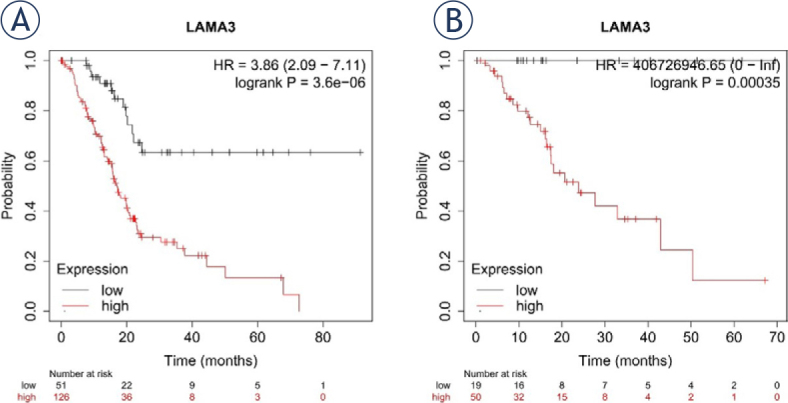
The expression of laminin subunit alpha 3 (LAMA3) for prediction of overall survival (OS) and relapse free survival (RFS) in patients with pancreatic ductal adenocarcinoma (PDAC). OS **(A)**, RFS **(B)**.

Survival curves were generated using the Kaplan–Meier Plotter database. Elevated LAMA3 expression was positively associated with poorer OS (Figure 2A, hazard ratio [HR] = 3.86, *P* < 0.001) and relapse-free survival (RFS) (Figure 2B, HR = 406726946.65, *P* < 0.001). These results demonstrate that high LAMA3 expression is associated with an unfavorable prognosis in patients with PDAC.

### Clinical characteristics of patients with PDAC

After meticulous filtering according to the inclusion and exclusion criteria, 117 patients with PDAC were included in this study. The baseline characteristics of the patients are summarized in Table 1. The mean age of all patients was 62.43 ± 9.33 years, with males accounting for 73 (62.3%) of the total population. Pancreaticoduodenectomy and distal pancreatectomy were performed in 69 and 48 patients, respectively. In our cohort, 61 postsurgical patients received chemotherapy, 3 patients received radiotherapy, 6 patients received immunotherapy, and 4 patients received interventional therapy. Based on the immunohistochemical results of LAMA3 expression levels, the patients were categorized into groups with high or low expression. Notably, 62 patients showed elevated LAMA3 expression. The univariate analyses of the two cohorts are presented in Table 1.

**TABLE 1.** Characteristics of all patients

**Characteristics**	**All**	**LAMA3 expression**		

		**High (n = 62)**	**Low (n = 55)**	***P-* value**
Age(year), mean±SD	62.43 ± 9.33	62.19 ± 9.38	62.69 ± 9.36	0.775
Sex, n (%)				0.615
Male	73 (62.4)	40	33	
Female	64 (37.6)	22	22	
Tumor location, n (%)				0.054
Head	70 (59.8)	32	38	
Body and tail	47 (40.2)	30	17	
Tumor size, n (%)				**0.007**
≤ 2 cm	5 (4.3)	2	3	
> 2 cm and ≤ 4 cm	81 (69.2)	37	44	
> 4 cm	31 (26.5)	23	8	
Histological grade, n (%)				0.810
G1	31 (26.5)	17	14	
G2–3	86 (73.5)	45	41	
TNM stage, n (%)				**0.002**
I–II	92 (78.6)	42	50	
III–IV	25 (21.4)	20	5	
Perineural invasion, n (%)				0.921
Yes	91 (77.8)	48	43	
No	26 (22.2)	14	12	
Vascular invasion, n (%)				0.340
Yes	37 (31.6)	22	15	
No	80 (68.4)	40	40	
Lymph node metastasis, n (%)				0.255
Yes	49 (41.9)	29	20	
No	68 (58.2)	33	35	
Liver metastasis, n (%)				**0.005**
Yes	45	30	15	
No	72	29	43	
CEA (ng/ml)				0.392
≤ 12	108 (92.3)	56	52	
> 12	9 (7.7)	6	3	
CA19-9 (U/ml)				0.395
≤ 282	74 (63.2)	37	37	
> 282	43 (36.8)	25	18	
Surgical modalities, n (%)				**0.036**
Pancreaticoduodenectomy	69 (59)	31	38	
Distal pancreatectomy	48 (41)	31	17	
Postoperative chemotherapy, n (%)				0.77
Yes	60	40	20	
No	57	41	16	
Postoperative radiotherapy, n (%)				0.063
Yes	3	2	1	
No	114	79	35	
Immunotherapy, n (%)				0.068
Yes	6	4	2	
No	111	77	34	
Interventional therapy, n (%)				0.374
Yes	4	4		
No	114	77	36	

CEA = carcinoembryonic antigen; CA19-9 = carbohydrate antigen 19-9; LAMA3 = laminin subunit alpha 3

Increased LAMA3 expression correlated with large tumor size (P = 0.007), and the degree of LAMA3 expression was associated with different TNM stages (P = 0.002). In addition, LAMA3 expression was higher in tumor tissue from patients with PDAC and liver metastases than those without liver metastases (P = 0.005). In the two groups, the surgical modalities used were significantly different (P = 0.036), but there were no significant differences in age, gender, tumor location, histological grade, perineural invasion, vascular invasion, lymph node metastasis, CEA and CA19-9 levels, and adjuvant systemic therapy (P > 0.05).

### Heightened expression of LAMA3 in PDAC tissues relative to adjacent tissues

**FIGURE 3.**
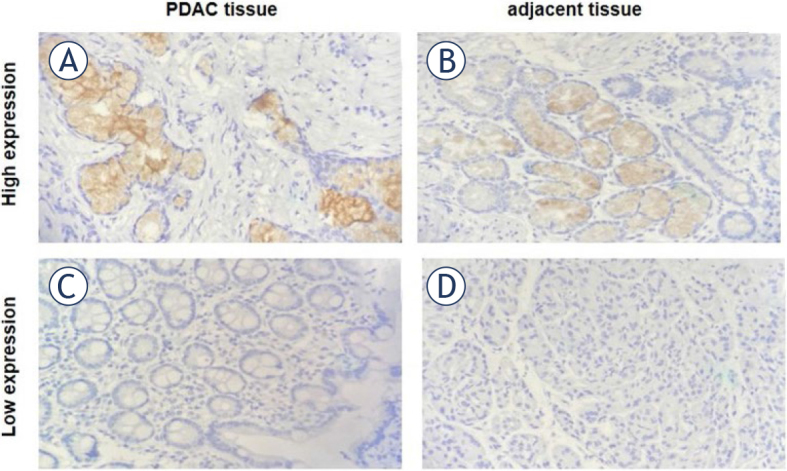
Representative immunohistochemical staining of laminin subunit alpha 3 (LAMA3) in pancreatic ductal adenocarcinoma (PDAC) and adjacent normal tissue. High expression of LAMA3 in PDAC tissue **(A)** compared with adjacent tissue **(B)**. Low expression of LAMA3 in PDAC tissue **(C)** compared with adjacent tissue **(D)**. Magnification, ×400

Immunohistochemistry was performed to measure LAMA3 expression in PDAC and adjacent normal tissues. LAMA3 staining was almost undetectable in normal tissues, and protein intensity was negative (Supplementary Table 1, Figure 3). Conversely, moderate staining and a robust intensity of LAMA3 protein expression were observed in PDAC tissues. The results demonstrated that LAMA3 expression was significantly higher in carcinoma specimens than in the adjacent tissues (*P* < 0.001).

### Superior expression of LAMA3 in PDAC tumor tissues exhibiting liver metastasis

**FIGURE 4.**
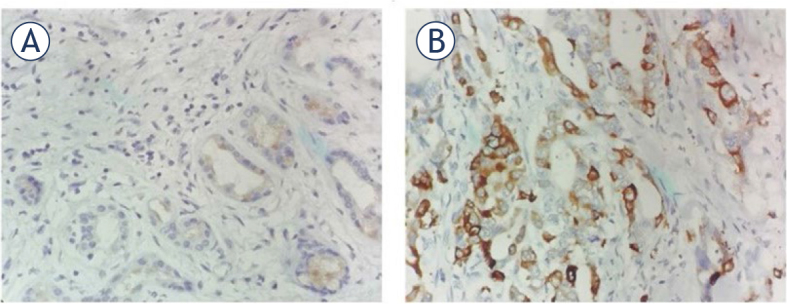
Representative immunohistochemical staining of laminin subunit alpha 3 (LAMA3) in pancreatic ductal adenocarcinoma (PDAC) with and without liver metastasis. Low expression of LAMA3 in PDAC tissues **(A)** without liver metastatic. High expression of LAMA3 in PDAC tissues **(B)** with liver metastasis. Magnification, ×400.

All patients with PDAC were categorized into two groups based on the emergence or absence of liver metastasis postoperatively (Table 2). Univariate analysis showed that histological grade (*P* = 0.001), TNM stage (*P* = 0.013), and vascular invasion (*P* = 0.018) were significantly associated with liver metastasis. Immunohistochemistry was used to assess LAMA3 expression in patients with PDAC with or without liver metastasis. LAMA3 expression in tumor tissues from patients with PDAC and liver metastasis was significantly higher than in those without liver metastasis (*P* = 0.005). Representative immunohistochemical images are shown in Figure 4. Age, sex, tumor location, tumor size, lymph node metastasis, perineural invasion, and serum CEA and CA19-9 levels were not associated with the development of liver metastasis. Significant factors from the univariate analysis, as shown in Table 2, were incorporated into the multivariate logistic regression analysis (Supplementary Table 2). The results showed that histological grade and LAMA3 expression were independently associated with liver metastasis.

**TABLE 2.** Univariate analysis of clinicopathological characteristics in patients with pancreatic ductal adenocarcinoma with and without liver metastasis

**Characteristics**	**Liver metastasis (n = 45)**	**Without liver metastasis (n = 72)**	***P*-value**
Age (year), M (IQR)	63 (58–69)	63 (56–68)	0.814
Sex, n			0.717
Male	29	44	
Female	16	28	
Tumor location, n			0.456
Head	25	45	
Body and tail	20	27	
Tumor size, n			0.240
≤ 2 cm	2	3	
> 2 cm and ≤4 cm	28	53	
> 4 cm	15	16	
Histological grade, n			**0.001**
G1	20	11	
G2–3	25	61	
TNM stage, n			**0.013**
I–II	30	62	
III–IV	15	10	
Perineural invasion, n			0.170
Yes	38	53	
No	7	19	
Vascular invasion, n			**0.018**
Yes	20	17	
No	25	55	
Lymph node metastasis, n			0.110
Yes	23	26	
No	22	46	
CEA (ng/ml)			0.701
≤ 12	41	67	
> 12	4	5	
CA19-9 (U/ml)			0.832
≤ 282	29	45	
> 282	16	27	
LAMA3 expression, n			**0.005**
High	30	29	
Low	15	43	

CEA = carcinoembryonic antigen; CA19-9 = carbohydrate antigen 19-9; LAMA3 = laminin subunit alpha 3

### High LAMA3 expression correlates with poor PDAC prognosis

**FIGURE 5.**
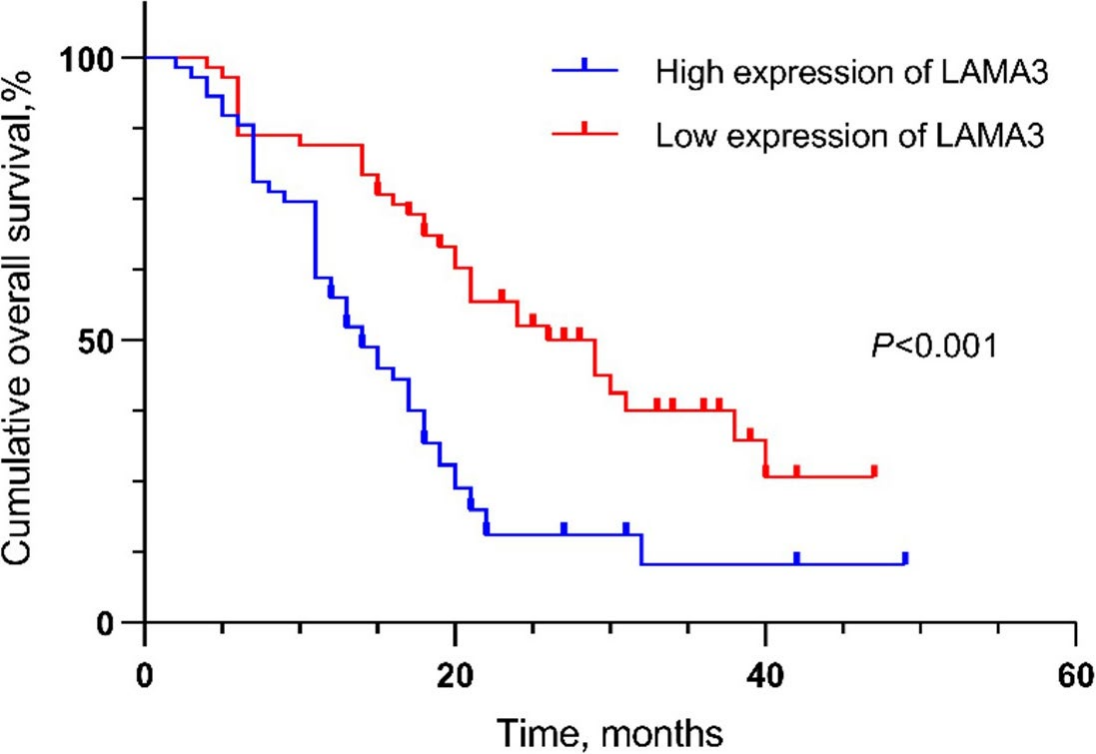
The expression of laminin subunit alpha 3 (LAMA3) for prediction of overall survival (OS) in patients with Pancreatic ductal adenocarcinoma (PDAC). Survival analysis was carried out with Kaplan-Meier and checked by log-rank test.

The median survival times of patients with low and high LAMA3 expressions were 29 and 14 months, respectively. The 1-, 2-, and 3-year survival rates of the high-expression group (n = 62) were 58.1%, 14.5%, and 4.8%, respectively. Conversely, those in the low-expression group (n = 55) were 90.9%, 47.2%, and 16.4%, respectively. Using Kaplan–Meier curves, high LAMA3 expression in PDAC was associated with poor OS, suggesting an unfavorable prognosis (P < 0.001) (Figure 5).

Univariate analysis suggested that tumor size, TNM stage, liver metastasis, and LAMA3 expression had a significant prognostic influence on OS (Table 3). Multivariate survival analysis revealed that LAMA3 expression (HR, 2.016; 95% confidence interval [CI], 1.257–3.234; *P* = 0.004) and liver metastasis (HR, 2.284; 95% CI, 1.426–3.657; *P* = 0.001) were independent predictive factors of OS.

**TABLE 3.** Univariate and multivariate Cox proportional hazard regression analyses of overall survival

**Variables**	**Univariate analysis**		**Multivariate analysis**	

	**HR (95% CI)**	***P*-value**	**HR 95% CI)**	***P*-value**
Age	1.009 (0.985–1.035)	0.459	1.009 (0.983–1.035)	0.512
Sex	1.346 (0.850–2.131)	0.205	0.696 (0.435–1.112)	0.696
Tumor location (head vs. body and tail)	0.895 (0.573–1.399)	0.628	0.780 (0.474–1.283)	0.327
Tumor size				
≤ 2 cm vs. > 4 cm	0.543 (0.186–1.583)	0.263	0.626 (0.191–2.054)	0.440
> 2 cm and ≤ 4 cm vs. > 4 cm	0.607 (0.373–0.987)	**0.044**	0.637 (0.337–1.204)	0.165
Histological grade (G1 vs. G2–3)	1.263 (0.778–2.049)	0.345	1.378 (0.797–2.383)	0.251
TNM stage (I–II vs. III–IV)	0.374 (0.227–0.615)	**< 0.001**	1.505 (0.855–2.647)	0.157
Perineural invasion (yes vs. no)	0.803 (0.464–1.389)	0.432	1.158 (0.611–2.196)	0.652
Vascular invasion (yes vs. no)	0.693 (0.442–1.085)	0.109	0.912 (0.529–1.572)	0.741
Lymph node metastasis (yes vs. no)	0.796 (0.513–1.235)	0.308	0.883 (0.548–1.423)	0.609
Liver metastasis (yes vs. no)	0.364 (0.231–0.574)	**< 0.001**	2.284 (1.426–3.657)	**0.001**
CEA (≤12 vs. >12)	0.512 (0.256–1.026)	0.059	1.622 (0.788–3.340)	0.189
CA19-9(≤282 vs. >282)	0.969 (0.613–1.533)	0.894	0.963 (0.560–1.655)	0.891
LAMA3 (low vs. high)	0.407 (0.259–0.641)	**< 0.001**	2.016 (1.257–3.234)	**0.004**

CA19-9 = carbohydrate antigen 19-9; CEA = carcinoembryonic antigen; CI = confidence interval; HR = hazard ratio; LAMA3 = laminin subunit alpha 3

## Discussion

Pancreatic cancer is a highly aggressive neoplasm of the digestive system and is characterized by a mortality rate equal to its incidence rate. Its strong invasiveness and early metastasis render approximately 80% of patient ineligible for surgical intervention at the time of diagnosis. This results in a 5-year survival rate < 10%. Current treatment approaches for PDAC include surgical resection combined with chemotherapy, radiation therapy, interventional therapy, and immunotherapy. Even in patients with resectable localized tumors, the postoperative 5-year survival rate remains approximately 20%.^12^ The major contributor to the high mortality rate of PDAC is its propensity for early metastasis, which poses a significant challenge in clinical management. Consequently, there is an urgent need to identify additional predictive biomarkers to enhance the risk stratification of patients with PDAC.

LAMA3, a member of the laminin family, plays a pivotal role in cellular processes by interacting with integrins on the cell membrane and participating in the intracellular signal transduction pathways. Recent studies have implicated elevated LAMA3 expression in various types of tumors, where it appears to promote cell proliferation, apoptosis, and tumor progression by modulating signal transduction pathways.^7, 8, 9^ Zboralski *et al.* demonstrated the simultaneous functional inactivation of the tumor suppressor mothers against decapentaplegic homolog 4 (SMAD4) and invasive growth of tumors in rectal and pancreatic cancers. Laminin 332 (LM-332) is the target of SMAD4, a positive transcriptional regulator that promotes the transcription of LAMA3, LAMB3, and LAMC2 genes encoding LM-332. SMAD4 mediates transcriptional activity through distinct molecular mechanisms associated with the LAMA3, LAMB3, and LAMC2 promoters.^13^ Additionally, Huang *et al.* highlighted the increased expression of LAMA3 protein in PDAC tumor cells relative to that in normal pancreatic cells. They further showed that high LAMA3 expression promoted the proliferation, migration, and invasion of PDAC tumor cells.^5^ However, the effect of LAMA3 on liver metastasis in PDAC has not been fully elucidated. This study aimed to clarify the expression profile of LAMA3 in PDAC and investigate its potential association with liver metastasis in patients diagnosed with unresectable PDAC.

In this study, we integrated data from the TCGA database with four independent datasets from the GEO database as validation cohorts and found that LAMA3 expression was upregulated in PDAC, validating previous study findings.^14^ Furthermore, through the analysis of clinical samples, we observed that LAMA3 was overexpressed in pancreatic carcinoma tissues compared with adjacent noncancerous tissues. When we analyzed clinicopathological data from the UALCAN database, we found that LAMA3 expression levels correlated with the histological grade of tumors in patients with PDAC. However, our analysis of clinical data revealed that high LAMA3 expression was associated with larger tumor size, advanced TNM stage, and liver metastasis. This discrepancy may be due to the inherent bias from our relatively small sample size. Jun *et al.* demonstrated that overexpression of the α3, β3, and γ2 chains of LM-332 might play a crucial role in the progression and prognosis of PDAC.^15^ Based on these findings, we evaluated the prognostic value of LAMA3 expression in patients with PDAC. Using the Kaplan–Meier Plotter dataset, which incorporates data from the GEO, European Genome-phenome Archive, and TCGA databases, we found that high LAMA3 expression was strongly associated with worse OS and RFS in patients with PDAC. Additionally, we followed up 117 patients with PDAC and observed that high LAMA3 expression in PDAC was correlated with poor OS, indicating an overall poor prognosis. Univariate and multivariate Cox regression analyses further demonstrated that LAMA3 was an independent predictive factor for mortality in patients with PDAC. In conclusion, these results strongly support that the expression of LAMA3 can serve as a robust prognostic biomarker of PDAC.

Metastasis, the primary cause of cancer-related mortality, continues to be an area of limited understanding regarding its cellular and molecular mechanisms.^16^ Various studies have implicated LAMA3 in different mechanisms of metastasis. Shu *et al.* demonstrated that the overexpression of LINC00936 hindered ovarian cancer progression by competitively binding to miR-221-3p and modulating LAMA3 expression.^7^ Moreover, Xu *et al.* reported that LINC00628 could obstruct cell proliferation, invasion, migration, and apoptosis while reducing drug resistance in lung adenocarcinoma cells by downregulating the methylation of the LAMA3 promoter^8^. Kinoshita *et al.* demonstrated that miRNA-218 modulated the focal adhesion pathway, thereby impeding tumor cell invasion and metastasis.^9^ The liver, which serves as the primary blood-borne drainage site for related organs, such as the portal vein system, colon, and pancreas, is crucial for distant metastasis in patients with PDAC.17 To gain a deeper understanding of the correlation between LAMA3 and liver metastasis in PDAC, we examined independent risk factors associated with liver metastasis. Univariate analysis revealed that histological grade, TNM stage, vascular invasion, and high LAMA3 expression were significantly associated with liver metastasis. Multivariate logistic regression analyses demonstrated that LAMA3 and histological grade were independent predictive factors for liver metastasis in patients with PDAC. These findings indicate that poor differentiation and high LAMA3 expression are correlated with an increased risk of metastasis.

This study has both strengths and limitations, necessitating further investigations to confirm and expand our findings. One of the strengths of this study is the use of data from public databases combined with bioinformatics analysis. This approach allows the utilization of large amounts of data, thus increasing the reliability and statistical power of our findings. We complemented this database analysis with clinical data to verify our results by adding another validation layer. However, this study has some limitations. This study was entirely based on data from public databases; although we used clinical data to confirm our findings, future studies with larger sample sizes and varied population groups are required to further validate our results. We evaluated LAMA3 expression in PDAC tissues using immunohistochemistry, which, although a common and reliable technique, only provides a snapshot of LAMA3 expression and does not provide functional information. Therefore, additional functional experiments are required to better understand the role of LAMA3 in PDAC. Finally, our study did not fully explore the mechanism by which LAMA3 promotes liver metastasis in PDAC. Understanding these mechanisms requires a series of in-depth molecular and cellular biology studies involving in vitro and in vivo models. By identifying and understanding the precise mechanisms involved, new potential therapeutic targets for PDAC can be identified.

This retrospective analysis suggests that LAMA3 may serve as a potential biomarker for predicting the prognosis of patients with PDAC. The increase in LAMA3 expression in PDAC tissues and its association with liver metastasis further underscore its potential role in disease progression. While these findings provide important insights, they also highlight the need for further studies. Understanding the specific mechanisms by which LAMA3 contributes to PDAC progression and liver metastasis may help uncover new therapeutic targets, potentially leading to more personalized treatment strategies.

Increased LAMA3 expression is associated with poor prognosis and liver metastasis in patients with PDAC. Our results indicate that LAMA3 can be a novel predictor of poor prognosis in patients with PDAC and liver metastasis, and LAMA3 may be a promising candidate for targeted therapy for PDAC liver metastasis.

## Supplementary Material

Supplementary Material Details
