# Supplementary material for: Correlation of laminin subunit alpha 3 expression in pancreatic ductal adenocarcinoma with tumor liver metastasis and survival
Source: Radiol Oncol. 2024 Mar 7;58(2):234–42. doi: 10.2478/raon-2024-0020 (PMC11165973; doi:10.2478/raon-2024-0020)
Supplement: Supplementary file 1 — Supplementary Material Details [file raon-2024-0020-sm.pdf]

sciendo Radiology and Oncology | Ljubljana | Slovenia | www.radioloncol.com

RADIOLOGY AND ONCOLOGY

Correlation of laminin subunit alpha 3 expression in pancreatic ductal adenocarcinoma with tumor liver metastasis and survival

Yueyi Xing, Xue Jing, Gong Qing, Yueping Jiang

doi: 10.2478/raon-2024-0020

SUPPLEMTARY TABLE 1. Laminin subunit alpha 3 expression levels in pancreatic cancer and adjacent tissues

	High	Low	*P*-value
Pancreatic cancer tissues	59 (50.4%)	58 (49.6%)	< 0.001
Adjacent tissues	1 (1.7%)	59 (98.3%)	

SUPPLEMTARY TABLE 2. Multivariate logistic regression analysis of clinicopathological characteristics in patients with pancreatic ductal adenocarcinoma with and without liver metastasis

Variables	B	Standard error	Wald	*P*-value	Odds ratio	95% confidence interval
Histological grade	1.455	0.484	9.024	**0.003**	0.233	0.090–0.603
Tumor-node-metastasis stage	0.738	0.518	2.032	0.154	2.093	0.758–5.776
Vascular invasion	0.553	0.458	1.457	0.227	0.575	0.234–1.412
Laminin subunit alpha 3 expression	1.001	0.451	4.923	**0.027**	2.720	1.124–6.582

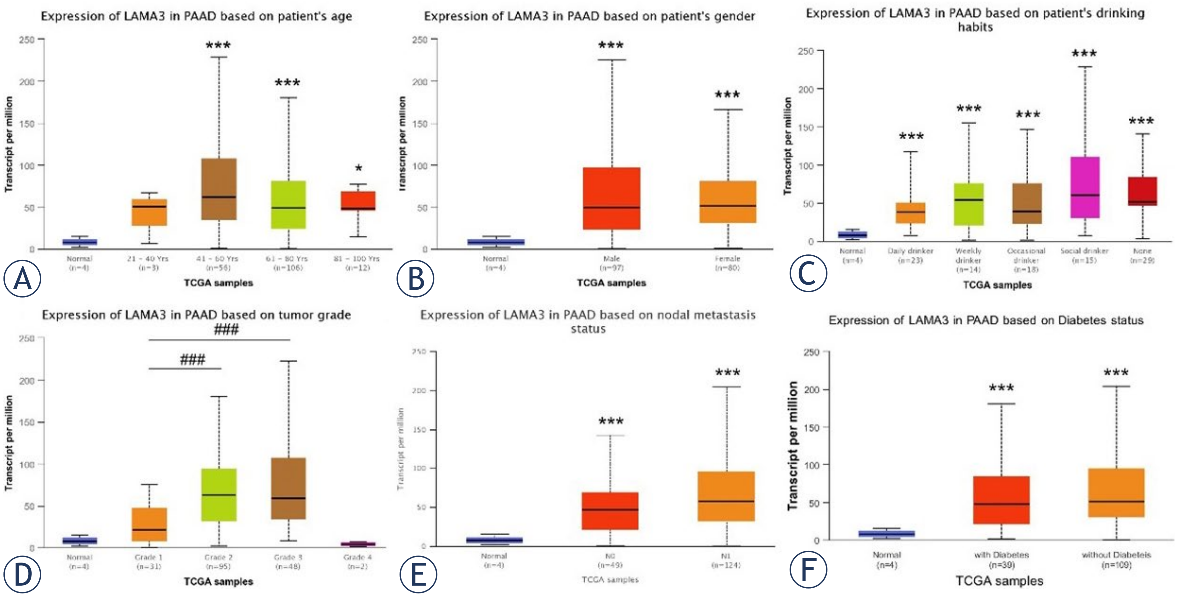

SUPPLEMENTARY FIGURE 1. Clinicopathological analysis of laminin subunit alpha 3 (LAMA3) in pancreatic ductal adenocarcinoma (PDAC) by University of ALabama at Birmingham CANcer (UALCAN). Age **(A)**. Gender **(B)**. Drinking habits **(C)**. Tumor grade **(D)**. Nodal metastasis **(E)**. Diabetes status **(F)**.

Compared with normal group: *P < 0.05, **P < 0.01 and ***P <0.001. Multiple comparisons: ###P < 0.001.
